# Exclusion of NUMB Exon12 Controls Cancer Cell Migration through Regulation of Notch1-SMAD3 Crosstalk

**DOI:** 10.3390/ijms23084363

**Published:** 2022-04-14

**Authors:** Zheng Zhan, Ningyang Yuan, Xue You, Kai Meng, Rula Sha, Zhenzhen Wang, Qian Peng, Zhiqin Xie, Ruijiao Chen, Ying Feng

**Affiliations:** 1 CAS Key Laboratory of Nutrition, Metabolism and Food Safety, Shanghai Institute of Nutrition and Health, University of Chinese Academy of Sciences, Chinese Academy of Sciences, Shanghai 200031, China; zzhan@sibs.ac.cn (Z.Z.); yuanningyang2018@sibs.ac.cn (N.Y.); sharula2018@sibs.ac.cn (R.S.); wangzhenzhen2019@sibs.ac.cn (Z.W.); pengqian2021@sibs.ac.cn (Q.P.); zqxie@sibs.ac.cn (Z.X.); 2 Collaborative Innovation Center for Birth Defect Research and Transformation of Shandong Province, Jining Medical University, Jining 272067, China; youxue19910@163.com (X.Y.); mengkai521888@126.com (K.M.)

**Keywords:** NUMB, isoforms, cell migration, Notch1, SMAD3

## Abstract

NUMB is an endocytic adaptor protein that contains four isoforms (p65, p66, p71 and p72) due to alternative splicing regulation. Here, we show that NUMB exon12 (E12)-skipping isoforms p65/p66 promote epithelial to mesenchymal transition (EMT) and cancer cell migration in vitro, and facilitate cancer metastasis in mice, whereas E12-included p71/p72 isoforms attenuate these effects. Mechanistically, p65/p66 isoforms significantly increase the sorting of Notch1 through early endosomes (EEs) for enhanced Notch1 activity. In contrast, p71/p72 isoforms act as negative regulators of Notch1 by ubiquitylating the Notch1 intracellular domain (N1ICD) and promoting its degradation. Moreover, we observed that the interaction between N1ICD and SMAD3 is important for their own stabilization, and for NUMB-mediated EMT response and cell migration. Either N1ICD or SMAD3 overexpression could significantly recuse the migration reduction seen in the p65/p66 knockdown, and Notch1 or SMAD3 knockdown rescued the migration advantage seen in the overexpression of p66. Taken all together, our study provides mechanistic insights into the opposite regulation of Notch1-SMAD3 crosstalk by NUMB isoforms and identifies them as critical regulators of EMT and cancer cell migration.

## 1. Introduction

Epithelial to mesenchymal transition (EMT) is a highly coordinated process by which epithelial cells lose cell–cell connections and cell polarity to become invasive mesenchymal phenotypes [1,2]. The phenotypic changes involve the activation of EMT transcription factors such as Snail and Slug, the downregulation of the epithelial marker E-cadherin, and the concurrent upregulation of the mesenchymal markers N-cadherin and fibronectin. Normal EMT program plays crucial roles during embryonic development and wound healing. However, the aberrant activation of EMT has emerged as a central driver of tumor malignancy [3,4,5].

EMT could be induced by a variety of molecules and multiple signaling pathways, including TGF-β, Notch and Wnt/β-catenin. TGF-β signaling has been recognized as a major driver for tumor metastasis and progression [6,7]. TGF-β-induced EMT is characterized by the repression of E-cadherin and the induction of N-cadherin, which is crucial to the malignant phenotype of various cancer cells. Distinct signaling pathways are involved in EMT mediated by TGF-β, among which SMAD3 is a key mediator of EMT and usually overexpressed in numerous cancers. Notch signaling is a highly conserved pathway that is activated by ligand binding and subsequent proteolytic cleavages of Notch to release NICD. The following nuclear translocation of NICD drives Slug and Snail expression, which promotes EMT. The crucial role of Notch1 in EMT was first reported during mouse cardiac valve formation, where the inactivation of Notch1 causes severely impaired EMT and pericardial edema [8]. Meanwhile, numerous studies showed that abnormal Notch signaling has been observed in human carcinomas, and enhanced levels of Notch1 are correlated with increased metastasis and poor survival [9,10,11]. More importantly, TGF-β signaling and Notch signaling communicate with each other during the induction of EMT [11]. For example, the chemical inactivation of Notch1 could block TGF-β-induced EMT, indicating the integration of the two signalings in the EMT process [12].

Human NUMB is the homologue of the numb protein that was initially discovered in *Drosophila melanogaster* as an adaptor protein. NUMB is identified as an endocytic matrix protein and participates in endocytic trafficking of a number of key molecules, for example, beta-amyloid precursor protein (APR) and Notch1. NUMB controls the intracellular trafficking of APP for membrane recycling and thus may be involved in APP metabolism and Alzheimer’s disease pathogenesis [13]. Changes in NUMB expression alter the dynamics of Notch1 trafficking and result in its degradation, indicating that NUMB functions as an inhibitor of Notch1 [14,15]. Meanwhile, *NUMB* pre-mRNA is subject to alternative splicing regulation, which generates four isoforms p65, p66, p71, and p72 [16]. The isoforms differ in the inclusion or exclusion of *NUMB* exon 6 (E6) in the N-terminal phosphotyrosine binding (PTB) domain or exon 12 (E12) in the proline rich region (PRR). Previous studies reported that these isoforms have redundant but distinct functions in various cellular processes such as cell proliferation, differentiation and tumorigenesis [17]. It remains to be characterized how the NUMB isoforms regulate cancer cell migration and cancer metastasis. 

In this study, we find that *NUMB* E12-skipping p65/p66 isoforms are mainly expressed in highly migratory cancer cell lines, whereas the E12-included p71/p72 isoforms are predominant in low migration cancer cell lines. This strongly indicates that the expression of p65/p66 isoforms is closely related the migratory behavior of cancer cells. Through isoform-specific knockdown and isoform-specific expression experiments, we demonstrate that the p65/p66 isoforms promote EMT and the migration of cancer cells, whereas p71/p72 isoforms attenuate these effects. Our data further reveal that NUMB p65/p66 isoforms increase Notch1 activity, and N1ICD works in concert with SMAD3 to promote EMT and thus enhance cancer cell migratory abilities. By contrast, the p71/p72 isoforms inhibit Notch1 activity by ubiquitylating N1ICD, leading to EMT reversion and reduced cancer cell migration. Taken together, our data demonstrate that NUMB isoforms control cancer cell EMT and migration through the distinct regulation of Notch1-SMAD3 crosstalk. 

## 2. Results

### 2.1. Expression of NUMB E12-Skipping p65/p66 Isoforms Promotes Cancer Cell Migration

**Figure 1:**
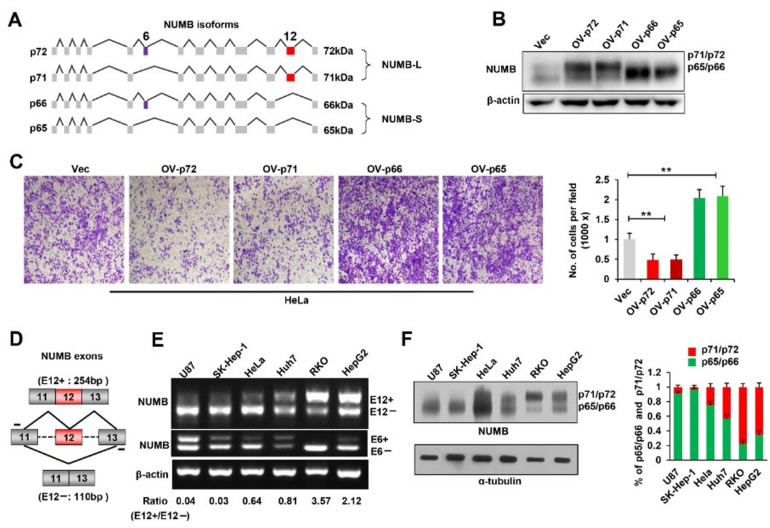
Expression of NUMB E12-skipping p65/p66 isoforms promotes cancer cell migration. (**A**) Schematic diagrams of *NUMB* splice variants. Constitutive exons are shown as grey boxes, whereas green and red boxes stand for alterative exons 6 and 12 (E6 and E12), respectively. (**B**) Overexpression of NUMB isoforms in HeLa cells. HeLa cells were transiently transfected with indicated expression plasmids. Whole-cell lysates were extracted and analyzed by Western blot. β-actin was used as a loading control. (**C**) Cell migration assay was performed with HeLa cells, as described in (**B**). Representative images of migratory cells stained with crystal violet are shown. Scale bar is 200 μm. Quantification was based on the three independent experiments, and results are shown as mean ± SD on the right. (**D**) Diagrams for detection of *NUMB* variants including or lacking E12 using RT-PCR. Primer pairs and product sizes for the two variants E12+ and E12− are shown. (**E**) Inclusion of *NUMB* E12 and E6 was examined in the six cancer cell lines by RT-PCR. Ratio for E12+/E12− is listed below the panel. (**F**) Whole-cell lysates were extracted from the six cancer cell lines described in (**E**) and subject to Western blot analysis using indicated antibodies. Qualification of NUMB isoforms is shown in the right bar graph. ** *p* < 0.01.

Human *NUMB* pre-mRNA contains two alternative exons (E6 and E12), whose different combination generates four isoforms, designated as p72, p71, p66 and p65, based on their molecular weight (Figure 1A). p71/ p72 isoforms include *NUMB* E12 in their transcripts, whereas the p65/p66 isoforms exclude E12. To address whether NUMB isoforms distinctly regulate cancer cell proliferation and invasion, we separately overexpressed four isoforms in HeLa cells (Figure 1B). The migration and proliferation of HeLa cells was investigated by the transwell migration assay and clonogenic survival assay, respectively. As shown, high levels of p71 and p72 isoforms caused pronounced decrease in the migratory abilities of the cells, whereas the overexpression of p65 and p66 isoforms dramatically increased their migration potential, compared to control cells (Figure 1C), indicating that the inclusion/exclusion of *NUMB* E12 has opposite effects on the capacity of cancer cells to migrate. On the other hand, the exclusion of *NUMB* E12 did not affect cell proliferation, as p65 overexpression increased the colony-forming abilities of HeLa cells in a similar manner to that of p71/p72 isoforms (Supplementary Figure S1). 

Next, we designed a pair of primers to detect the inclusion status of *NUMB* E12 and E6 in various human cancer cell lines (Figure 1D). Cancer cell lines deriving from liver tumors (Huh7 and HepG2) and from colon carcinomas (RKO) exhibit an epithelial-like morphology with low migration potential. Cell lines derived from a glioma (U87), from a liver adenocarcinoma (SK-Hep-1) and from a cervical carcinoma (HeLa) show mesenchymal or fibroblastic morphology, which are more often associated with increased invasion. RT-PCR analysis revealed that the skipping of *NUMB* E12 was predominant in U87, SK-Hep-1 and HeLa cells, whereas the inclusion of *NUMB* E12 was the major event in Huh7, RKO and HepG2 cells (Figure 1E). However, *NUMB* E6 was mostly skipped in all the cancer cell lines examined (Figure 1E). Western blot analysis further confirmed that p65/p66 isoforms were mainly expressed in highly migratory U87, SK-Hep-1 and HeLa cells. By contrast, p71/p72 isoforms were dominant in other cancer cell lines (Figure 1F). These results strongly indicated that the inclusion/exclusion of *NUMB* E12 but not E6 was closely related to the migration potential of various cancer cell lines. 

### 2.2. p71/p72 Knockdown Increased Cancer Cell Migration by Promoting EMT whereas p65/p66 Knockdown Inhibited EMT and Cell Migration

**Figure 2:**
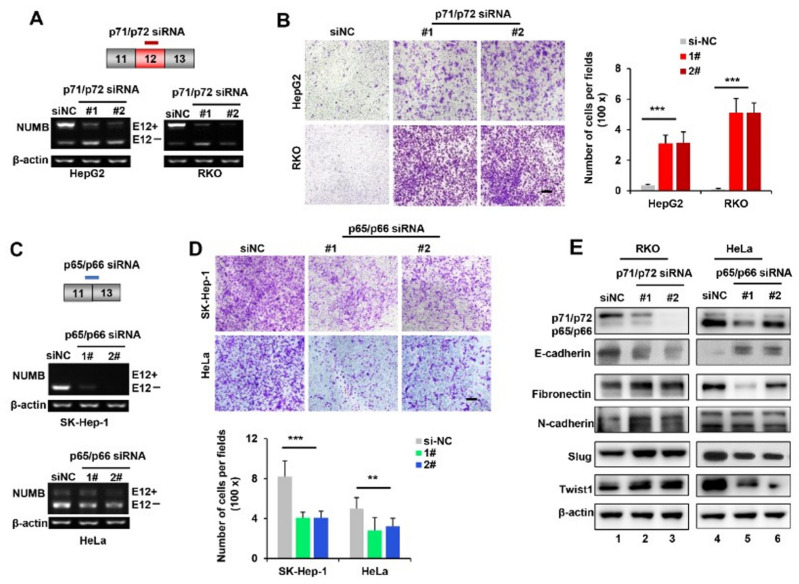
Knockdown of p71/p72 isoforms enhanced cancer cell migration by promoting EMT, whereas knockdown of p65/p66 isoforms inhibited cell migration by reversing EMT. (**A**) Diagram of siRNA targeted against the p71/p72 isoforms (top). Knockdown efficiency was assessed by RT-PCR analysis in HepG2 and RKO cells treated with control siRNA (siNC) and p71/p72 siRNAs (#1 and #2), respectively (bottom). (**B**) Transwell migration assays were performed using cells described in (**A**). Representative images of migratory cells stained with crystal violet were shown. Scale bar is 200 μm. The number of migratory cells was quantified, and results are shown as mean ± SD on the right bar graph. (**C**) Diagram of siRNA targeted against the p65/p66 isoforms (top). Knockdown efficiency was assessed by RT-PCR analysis in SK-Hep-1 and HeLa cells treated with siNC and p65/p66 siRNAs (#1 and #2), respectively (middle and bottom). (**D**) Transwell migration assays were performed with cells described in (**C**). Representative images of migratory cells stained with crystal violet were shown on the top. Scale bar is 200 μm. The number of migratory cells was quantified, and results are shown as mean ± SD on the bottom. (**E**) Western blot analysis was performed using whole-cell lysates isolated from RKO and HeLa cells described in (**A**,**C**). ** *p* < 0.01, *** *p* < 0.001.

We then examined how the specific knockdown of NUMB isoforms influenced cancer cell migration. To this end, we first designed two siRNAs that specifically targeted p71/p72 mRNA (Figure 2A, top). These two siRNAs displayed great efficacies of reducing p71/p72 transcripts in both HepG2 and RKO cells, as addressed by RT-PCR analysis (Figure 2A). qPCR analysis further revealed that they had no knockdown effects on p65/p66 isoforms (Supplementary Figure S2). Additionally, knockdown of p71/p72 isoforms significantly increased cancer cell migration abilities, compared to control siRNA (siNC)-transfected cells (Figure 2B). Then, we designed another two siRNAs specifically against the p65/p66 isoforms, which efficiently decreased the levels of p65/p66 isoforms in both SK-Hep-1 and HeLa cells, without effects on the levels of p71/p72 isoforms (Figure 2C, and Supplementary Figure S2). Not surprisingly, p65/p66 knockdown greatly decreased the migration potential of these two cancer cell lines (Figure 2D).

In agreement with above results, Western blot analysis further confirmed that p71/p72 depletion caused a significant reduction in E-cadherin and a marked increase in N-cadherin, fibronectin, Slug and Twist1 expression (Figure 2E, compare lanes 2–3 with lane 1). In contrast, opposite changes in EMT markers were observed in p65/p66 knockdown (compare lanes 5–6 with lane 4). Taken together, these findings strongly demonstrated that p65/p66 isoforms increased cancer cell migration by the upregulation of EMT, whereas the p71/p72 isoforms reversed EMT and thus inhibited cancer cell migration.

### 2.3. NUMB Isoforms Distinctly Controlled Tumor Migration and Metastasis in Mice 

**Figure 3:**
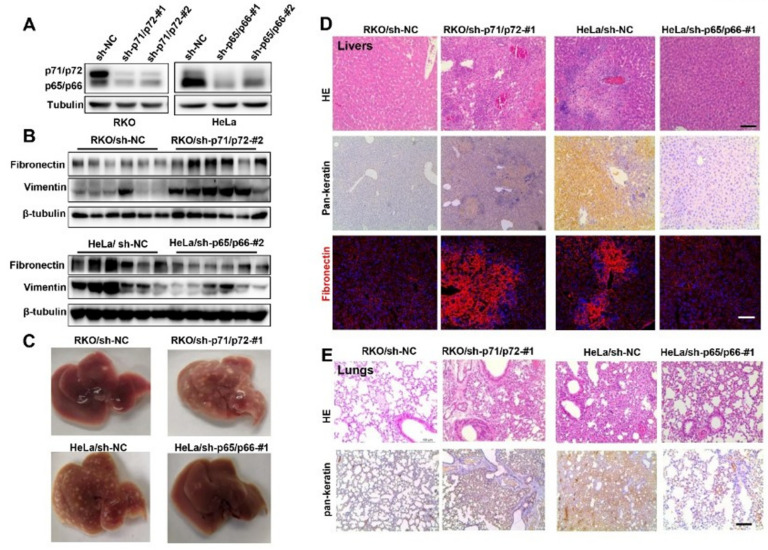
NUMB isoforms distinctly controlled tumor migration and metastasis in mice. (**A**) Western blot analysis of NUMB isoform knockdown in stable RKO cells or HeLa cells expressing sh-p71/72 (#1 and #2) or sh-p65/p66 (#1 and #2), respectively. (**B**) Stable RKO cells (sh-NC and sh-p71/p72-#2) and HeLa cells (sh-NC and sh-p65/p66-#2) were laterally abdominally injected into nude mice. Three weeks after the injection, livers were harvested, and protein extracts were examined by Western blotting with indicated antibodies. (**C**) Stable RKO cells (sh-p71/p72-#1 and sh-NC) and stable HeLa cells (sh-NC and sh-p65/p66-#1) were subcutaneously injected into nude mice. Mice were killed at four weeks after the injection, and representative images of livers were shown from the indicated groups. (**D**) Representative HE staining, IHC staining of pan-keratin and immunostaining of fibronectin were shown from indicated groups. Scale bar is 100 μm. (**E**) Representative HE staining and immunohistochemical staining images of lung sections prepared from the indicated groups. Scale bar is 100 μm.

We next wanted to investigate whether NUMB isoforms were distinctly implicated in cancer metastasis using a xenograft assay in nude mice. To this end, we generated stable RKO and HeLa cell lines stably expressing shRNAs against p71/p72 or p65/p66 isoforms, respectively. Western blot analysis revealed that the expression levels of isoform proteins were significantly decreased, compared to control cells (Figure 3A). qPCR analysis further conformed their specificity of knockdown effects (Supplementary Figure S3). Stable RKO or HeLa cells were subcutaneously injected into the right flanks of four-week-old nude mice. Preliminary trial with RKO/sh-p71/p72-2# cells, HeLa/p65/p66-#2 cells and control cells showed that livers could be the metastatic tissue of tumor cells, as addressed by Western blot analysis of Fibronectin and Vimentin proteins (Figure 3B). 

Next, we decided to give a thorough examination of the livers of mice after the injection of RKO/sh-p71/p72-#1, HeLa/sh-p65/p66-#1 and control cells. As shown in Figure 3C, the RKO/ sh-p71/p72-#1 group displayed numerous and large metastatic colonies, whereas only a few small colonies were detected in the livers of control mice (top panel). Quite the contrary, only a few small metastatic colonies were found in the livers of the HeLa/sh-p65/p66-#1 group, whereas the more severe tumor invasive foci were abundant in the control livers (bottom panel). HE staining showed many deeply stained infiltrating cells in the liver sections of p71/p72- knockdown group. IHC staining with pan-keratin confirmed the tumor characteristics of liver cells, which also displayed enhanced EMT through increased expression of Fibronectin in the RKO/sh-p71/p72-#1 group, compared to the control (Figure 3D, left panels). These data strongly demonstrated that the depletion of p71/p72 isoforms enhanced tumor metastasis in nude mice. On the other hand, HE and IHC staining were fully consistent with the decreased metastasis phenotypes of the HeLa/ sh-p65/p66-#1 group (Figure 3D, right panels). In addition, although there were no visible foci on the lungs, they also displayed increased and decreased metastasis phenotypes following p71/p72 and p65/p6 knockdown, respectively (Figure 3E). Together, we concluded that NUMB p71/p72 and p65/p66 isoforms displayed opposing influence on tumor migration and metastasis in vivo.

### 2.4. Notch1 Activation Is Required for p65/p66-Induced EMT and Tumor Cell Migration 

**Figure 4:**
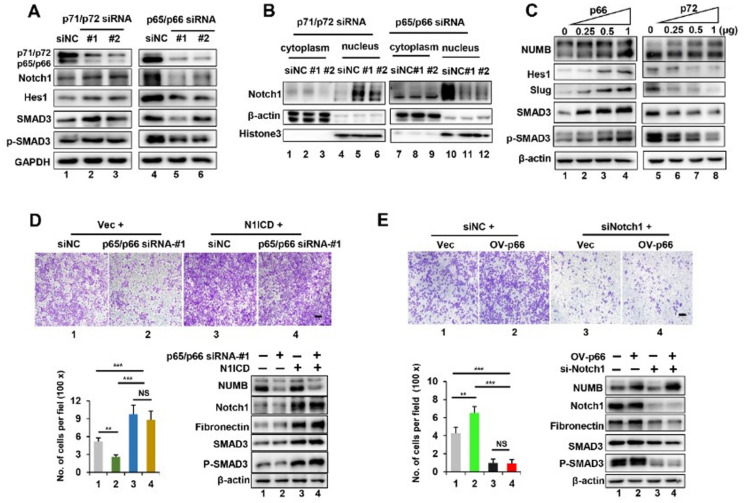
Notch1 activation is required for p65/p66-induced EMT and tumor cell migration. (**A**) RKO cells were transiently transfected with siNC and p71/p72 siRNA (#1 or #2), and HeLa cells were transiently transfected with siNC and p65/p66 siRNA (#1 or #2), followed by Western blotting analysis using indicated antibodies. GAPDH was used as loading controls. (**B**) Nuclear and cytoplasmic proteins were separately isolated from cells described in (**A**) and subjected to Western blotting analysis. β-actin and histone3 were used as loading controls. (**C**) RKO cells were transiently transfected with increasing amounts of a p66-expression plasmid, and HeLa cells were transfected with a p72-expression plasmid, followed by Western blot analysis with indicated antibodies. (**D**) HeLa cells were co-transfected with p65/p66 siRNA-#1 and an N1ICD-expression plasmid as indicated, followed by the cell migration assay. Representative images of migratory cells stained with crystal violet are shown (top). Scale bar is 200 μm. Quantification of migratory cells is shown as mean ± SD on the left bottom, and Western blot analysis with indicated antibodies is shown on the right bottom. (**E**) HeLa cells were co-transfected with anti-Notch1 siRNA (siNotch1) and a p66-expression plasmid as indicated, followed by the cell migration assay. Representative images of migratory cells stained with crystal violet are shown (top). Scale bar is 200 μm. Quantification of migratory cells is shown as mean ± SD in the bottom left, and Western blot analysis is shown in the bottom right. ** *p* < 0.01, *** *p* < 0.001, NS *p* > 0.05.

Next, we wanted to test whether NUMB isoforms differently influence the activity of Notch1 pathway. As shown in Figure 4A, protein levels of Notch1 and its target gene Hes1 were significantly increased in p71/p72-knockdown RKO cells, compared to control cells (compare lanes 2–3 with lane 1), which is consistent with previous reports that NUMB acts as an inhibitor of Notch1 signaling [18]. By contrast, decreased protein levels of Notch1 and Hes1 was observed in p65/p66-knockdown HeLa cells (compare lanes 5–6 with lane 4), which did not match previous reports. More importantly, an analysis of separated extracts further revealed that p71/p72 knockdown caused elevated nuclear localization of Notch1 (Figure 4B, compare lanes 5–6 with lane 4, and lanes 1–3), whereas p65/p66 knockdown resulted in a dramatic reduction in Notch1 in the nucleus (compare lanes 11–12 with lane 10, and lanes 7–9), indicating that NUMB isoforms have opposite regulation on the active form of Notch1 (N1ICD). Consistent with the knockdown results, p66 overexpression was sufficient to increase protein levels of Hes1 and Slug in RKO cells, whereas the overexpression of p72 significantly reduced their expression in HeLa cells (Figure 4C, compare lanes 2–4 with lane 1, lanes 6–8 with lane 5). Collectively, these results strongly indicated that p65/p66 isoforms acted as activators of Notch1 signaling, whereas p71/p72 isoforms repressed Notch1 activity. 

We reasoned that NUMB might mediate isoform-specific effects through the distinct regulation of Notch1 activity in tumor cells. For this purpose, N1ICD was over-expressed in HeLa cells in the presence or absence of p65/p66 deletion. As shown, the overexpression of N1ICD not only enhanced the migration abilities of HeLa cells, but also eliminated the reduction in migration ability seen in p65/p66 knockdown (Figure 4D, compare lane 1 and lane 3, lane 2 and lane 4). Additionally, N1ICD overexpression was correlated with increased protein levels of Fibronectin in HeLa cells, whether p65/p66 knockdown or not (Figure 4D, bottom panel on the right, compare lanes 3–4 with lane 2). These results indicated that the overexpression of N1ICD promoted an EMT response, which functions as the downstream of p65/p66 isoforms. Similarly, siRNA-mediated knockdown of Notch1 could robustly dampen the migration advantage seen in the overexpression of p66 (Figure 4E, top panel and left bottom panel: compare lane 4 with lane 2). Additionally, the downregulation of Notch1 decreased EMT response, as decreased Fibronectin levels were observed in the presence of p66 overexpression (Figure 4E, bottom panel on the right, compare lane 4 with lane 2). Taken together, these data demonstrated that activation of Notch1 is required for p65/p66-induced EMT and tumor cell migration. 

### 2.5. p71/p72 Isoforms but Not p65/p66 Isoforms Promoted Ubiquitylation of N1ICD, Leading to Its Degradation

**Figure 5:**
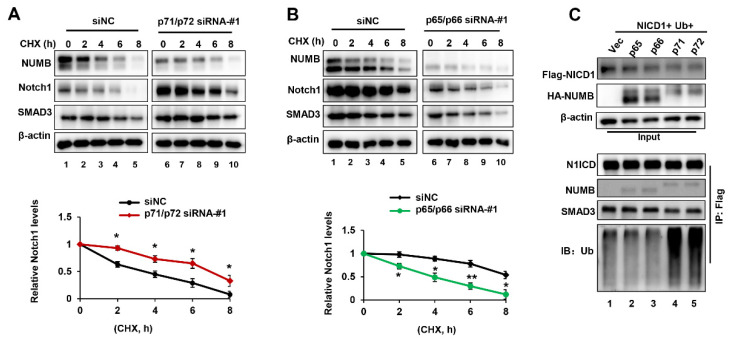
NUMB p71/p72 isoforms promoted ubiquitylation of N1ICD and its degradation. (**A**) RKO cells were transiently transfected with si-p71/72-#1 for 48 h, then incubated in the presence of cycloheximide (CHX, 50 μg/mL) for the indicated time course, followed by Western blotting analysis. Quantification of Notch1 proteins is shown at the bottom. (**B**) HeLa cells were transiently transfected with si-p65/p66-#1 for 48 h, then incubated in the presence of cycloheximide (CHX, 50 μg/mL) for the indicated time course, followed by Western blotting analysis. Quantification of Notch1 proteins is shown on the bottom. (**C**) 293T cells were co-transfected with Flag-N1ICD, Ubiquitin (Ub) and NUMB isoform expression plasmids. Whole-cell lysates were extracted and immunoprecipitated with anti-Flag antibodies followed by Western blotting analysis. Overall, 10% of whole cell lysates were used as input. * *p* < 0.05, ** *p* < 0.01.

NUMB proteins have been reported to be involved in Notch1 degradation [15]. To address this issue, we performed 8h cycloheximide (CHX) chase experiments using p71/p72-knockdown RKO cells and p65/p66-knockdown HeLa cells. As shown, the half-life of Notch1 proteins in p71/p72- knockdown cells was obviously longer than that in control cells (Figure 5A, compare lanes 6–10 and lanes 1–5), suggesting that p71/p72 isoforms promoted the degradation rate of Notch1. On the other hand, CHX treatment led to a significant decrease in the half-life of Notch1 in p65/p66-knockdown HeLa cells, compared to control cells (Figure 5B, compare lanes 6–10 and lanes 1–5), indicating that p65/p66 isoforms inhibited Notch1 degradation. 

Following the previous findings that the ubiquitylation of N1ICD is critical for proteasome-mediated degradation [15], next, we wanted to test whether NUMB isoforms played distinct roles in Notch1 ubiquitylation. To this end, we co-transfected 293T cells with Flag-N1ICD, Ubiquitin (Ub) expression plasmids and HA-NUMB isoforms. After co-immunoprecipitation (co-IP) and Western blot analysis, notably, we observed that the p71/p72 isoforms could produce robust Ub smears of N1ICD, whereas the p65/p66 isoforms exhibited little effect compared to the vector control (Figure 5C, compare lanes 4–5 with lanes 1–3). These findings demonstrate that p71/p72 not p65/p66 isoforms promoted ubiquitination of N1ICD and resulted in its degradation. 

### 2.6. NUMB Isoforms Distinctly Influence Post-Endocytic Trafficking of Notch1 

**Figure 6:**
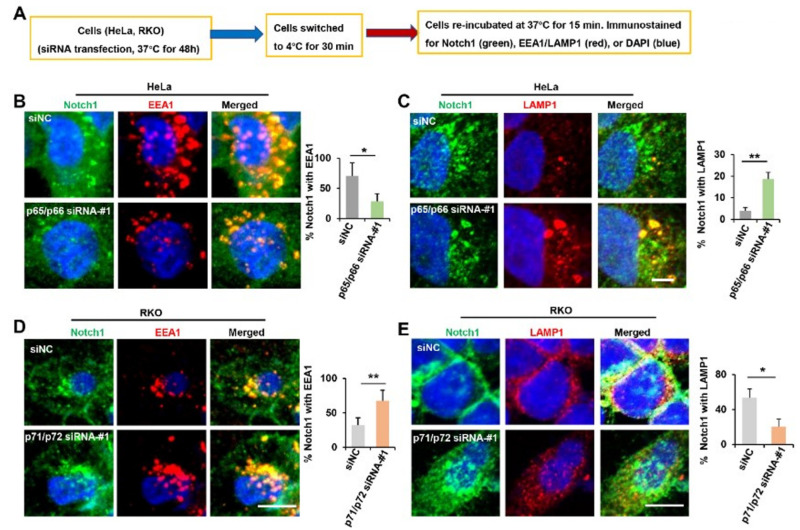
NUMB isoforms distinctly influence the post-endocytic trafficking of Notch1. (**A**) Diagram of cell treatment before immunostaining. (**B**,**C**) HeLa were treated with siNC and p65/p66 siRNA-#1, as described in (**A**), and stained with Notch1 (green), EEA1 (red) or LAMP1 (red). Representative confocal images were shown. Scale bar is 10 μm. Percentage of Notch1 colocalized with EEA1 or with LAMP1among the total amount of Notch1 is shown as mean ± SD in the right panel. (**D**,**E**) RKO cells were treated with siNC and p71/p72 siRNA-#1, as described in (**A**), and stained with Notch1 (green), EEA1 (red) or LAMP1 (red). Representative confocal images are shown. Scale bar is 10 μm. Percentage of Notch1 colocalized with EEA1 or with LAMP1 with the total amount of Notch1 is shown as mean ± SD on the right panel. * *p* < 0.05, ** *p* < 0.01.

Accumulating evidence suggests that the entry of Notch1 into the endosomal pathway and subsequent trafficking is closely linked to downstream signaling outcomes [14]. We next wanted to examine whether p65/p66 isoforms play distinct roles in the post-endocytic trafficking of Notch1 compared to p71/p72 isoforms. To this end, we performed double immunostaining to study the localization of Notch1 either in endosomes (EEs) or in the lysosome following p65/p66 depletion in HeLa cells (Figure 6A). As shown, most early endosome antigen 1 (EEA1)-positive EEs were localized in the perinuclear region. They were notably large clusters and overlapped with more than 70% of Notch1 signals in HeLa cells. In p65/p65-knockdown cells, EEs appeared as dispersed and small clusters, and only approximately 20% Notch1 signals were merged with EEA1(Figure 6B). On the other hand, only 5% of Notch1 signals were localized in the lysosome in control cells, which increased to approximately 20% following p65/p66 depletion (Figure 6C). These results indicated that knockdown of p65/p66 isoforms impaired the post-endocytic pathway of Notch1 and thus caused Notch1 accumulation in the lysosome.

In p71/p72-knockdown RKO cells, approximately 70% of Notch1 signals were accumulated in the concentrated EEs, compared to about 30% in control cells (Figure 6D). Meanwhile, p71/p72 knockdown also decreased Notch1 signals in the lysosome (Figure 6E). These findings indicated that p71/p72 knockdown resulted in increased post-endocytic trafficking of Notch1, either directing it recycling back to the cell membrane or promoting its nuclear localization. In conclusion, our findings strongly indicated that NUMB influenced the post-endocytic trafficking of Notch1 signaling in an isoform-dependent manner. 

### 2.7. Notch1 and SMAD3 Interaction Mediated NUMB’s Effects on Cancer Cell Migration

During the course of study, we also observed increased protein levels of SMAD3 and phosphorylated-SMAD3 (p-SMAD3) in both p71/p72-knockdown cells and p66-overxpressing cells (Figure 4A, compare lane 1 and lanes 2–3; Figure 4C, compare lane 1 and lanes 2–4). By contrast, their protein levels were significantly decreased in both p65/p66- knockdown cells and p72-overexpressing cells (Figure 4A, compare lane 4 and lanes 5–6; Figure 4C, compare lane 5 and lanes 6–8). 

**Figure 7:**
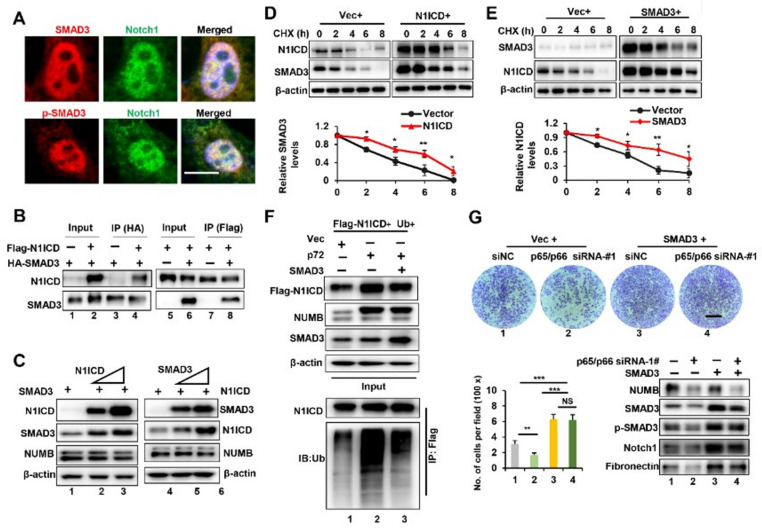
Notch1 and SMAD3 interactions medicated NUMB-induced EMT and cancer cell migration. (**A**) HeLa cells were transfected with the N1ICD expression plasmid, following by immunostaining with indicated antibodies. Representative confocal images are shown. Scale bar is 10 μm. (**B**) 293T cells were co-transfected with HA-SMAD3, Flag-N1ICD expression plasmids or control vector, as indicated. Whole-cell lysates were extracted and immunoprecipitated with anti-HA antibodies (lanes 1–4) or anti-Flag antibodies (lanes 5–8), followed by Western blot analysis. (**C**) 293T cells were co-transfected with a HA-SMAD3-expression plasmid and increasing amounts of Flag-N1ICD expression plasmids (lanes 1–3), or with a Flag-N1ICD expression plasmid and increasing amounts of HA-SMAD3 expression plasmids (lanes 4–6). Whole-cell lysates were extracted and subjected to Western blotting with indicated antibodies. Overall, 10% of whole cell lysates were used as input. (**D**) 293T cells were transiently transfected with the N1ICD expression plasmid for 48 h, followed by the addition of cycloheximide (CHX, 50 μg/mL), and Western blotting analysis. Quantification of SMAD3 proteins is shown at the bottom. (**E**) 293T cells were transiently transfected with the SMAD3 expression plasmid for 48 h, followed by the addition of cycloheximide (CHX, 50 μg/mL) and Western blotting analysis. Quantification of N1ICD proteins is shown at the bottom. (**F**) 293T cells were transiently co-transfected with indicated plasmids for 48 h. Whole-cell lysates were extracted and immunoprecipitated with anti-Flag antibodies followed by Western blotting analysis. Overall, 10% of whole cell lysates were used as input. (**G**) HeLa cells were co-transfected with p65/p66 siRNA-#1 and a SMAD3-expression plasmid, followed by the cell migration assay. Representative images of migratory cells stained with crystal violet were shown on the top. Scale bar is 360 μm. Quantification of migratory cells as mean ± SD was shown on the left bottom. Western blot analysis was shown on the right bottom. * *p* < 0.05, ** *p* < 0.01, *** *p* < 0.001, NS *p* > 0.05.

To further investigate interactions between SMAD3 and Notch1, we performed immunostaining of HeLa cells transfected with the N1ICD expression plasmid. The results demonstrated that SMAD3/p-SMAD3 is totally co-localized in the nucleus with N1ICD (Figure 7A). Using reciprocal IP, we confirmed that N1ICD and SMAD3 could interact with each other (Figure 7B). Moreover, protein levels of SMAD3 were significantly up-regulated due to elevated expression of N1ICD and vice versa, indicating that the interaction could increase their protein levels (Figure 7C). CHX chase experiments further demonstrated that the overexpression of N1ICD or SMAD3 significantly inhibited the degradation rate of SMAD3 or N1ICD in 293T cells (Figure 7D,E). 

As SMAD3 could stabilize N1ICD, next, we asked whether the overexpression of SMAD3 could prevent the p71/p72-mediated ubiquitination of N1ICD. To this end, we co-transfected 293T cells with Flag-N1ICD, Ub, SMAD3 and p72-expression plasmids. After co-IP and Western blot analysis, we observed that the overexpression of SMAD3 could significantly decreased p72-mediated ubiquitination of N1ICD (Figure 7F). Consistently, we further observed that SMAD3 overexpression significantly reduced the migration reduction seen in p65/p66 knockdown (Figure 7G). In addition, SMAD3 knockdown also rescued the migration advantage seen in p66 overexpression (Supplementary Figure S4). These results strongly demonstrated that cross-talk between SMAD3 and N1ICD play a critical role in mediating NUMB’s effects on cancer cell migration. 

## 3. Discussion

In this study, we have shown that NUMB p65/p66 isoforms promoted EMT and displayed a pro-metastatic function, whereas NUMB p71/p72 isoforms antagonized these effects. We further demonstrated that NUMB isoforms mediated these opposite effects through the distinct regulation of Notch1-SMAD3 complex. 

The antagonism between NUMB and Notch is well-established, by which NUMB antagonizes Notch1 by facilitating its ubiquitination and thereby promoting its degradation [14,15]. The silencing of NUMB in primary normal breast cells results in a significant increase in Hes-1 mRNA [19], and the aberrant activation of Notch signaling in human breast cancers was thought to be due to the loss of NUMB expression [18]. Although these findings did not distinguish between NUMB isoforms, the results were similar to the function of NUMB p71/p72 isoforms in this study. Our work not only confirmed the previous findings, but also further clarified that it was NUMB p71/p72 isoforms that promoted the ubiquitination of N1ICD and thus resulted in its degradation. Meanwhile, we also observed that Notch1 co-localized less with lysosomes upon NUMB p71/p72 knockdown. This possibly reflected that the p71/p72 isoforms also promoted Notch1 degradation in a lysosome-mediated manner. 

Importantly, emerging evidence suggests that Notch1 signaling mediates cancer cell invasion and metastasis by inducing EMT through the upregulation of Slug, which is involved in breast cancer metastasis in vitro and in vivo [20,21]. In line with these discoveries, our data further demonstrated that the loss of NUMB p71/p72 isoforms increased cancer cell migration by the activation of Notch1 activity and the upregulation of the EMT process, underscoring the importance of p71/p72 depletion in initiating Notch1-mediated EMT during cancer cell migration. 

Unexpectedly, our data revealed that NUMB p65/p66 isoforms have distinct functions compared to the p71/p72 isoforms. Firstly, they were mainly expressed in highly migratory cancer cell lines; by contrast, p71/p72 isoforms were dominant in low-migration cancer cell lines. Secondly, the p65/p66 isoforms increased cancer cell migration by the upregulation of the EMT process, whereas the p71/p72 isoforms attenuated these effects. Thirdly, the p65/p66 isoforms could significantly enhance Notch1 activity by facilitating the post-endocytic trafficking of Notch1 and concomitantly preventing its degradation, which is completely opposite to the antagonistic effect of traditional NUMB on Notch1. Consistent with our results, high levels of p65/p66 isoforms were reported to be expressed during BRCA1 depletion-driven EMT and two human basal-like breast cancer cell lines, further underscoring the importance of NUMB p65/p66 isoforms in mammary epithelial cells tumorigenesis [22]. Additionally, as NUMB E12 was located in the proline-rich region (PRR), the exclusion of E12 might change the PRR domain, resulting in functions of p65/p66 isoforms different from the p71/p72 isoforms containing intact PRR domains. 

A functional synergism was also observed between Notch1 and SMAD3 in the regulation of EMT and cancer cell migration. Indeed, there is increasing evidence demonstrating cross-talks between the Notch and TGF-β signaling pathways during various biological processes. SMAD3 interacted with N1ICD to induce IL-9 expression in T cells that participated in the regulation of the immune response [23]. Direct interaction between N1ICD and SMAD3 was involved in the regulation of Hes-1 expression in myogenic cells [24]. Importantly, we observed that SMAD3 and N1ICD interact with each other in the nucleus to enhance protein stability, so that the overexpression of SMAD3 prevented NUMB p71/p72-mediated ubiquitination of N1ICD. The effects of SMAD3 and Notch1 are almost the same in cancer cell migration, as knockdown of either of the two could decrease the migration advantage seen in the overexpression of NUMB-p66, and overexpression could rescue the migration reduction seen in p65/p66 knockdown. Together, our data demonstrated that the Notch1-SMAD3 interaction was important for NUMB isoforms-mediated cancer cell migration. 

In summary, our work has provided strong evidence that NUMB isoforms played opposing roles in the regulation of EMT, cancer cell migration and metastasis. The association of NUMB isoforms and Notch1-SMAD3 complex with clinical outcomes in human cancers should be further addressed in future studies. 

## 4. Materials and Methods

### 4.1. Cell Culture and Reagents

Cell lines HeLa, HepG2, SK-Hep-1, RKO, U87, Huh7 and 293T were purchased from the Chinses Academy of Science and grown in Dulbecco’s modified Eagle’s medium (DMEM, Gibco) supplemented with 10% fetal bovine serum (FBS, Gibco). All cell lines were incubated at 37 °C in a humidified 5% CO_2_ atmosphere. Antibodies including NUMB (2756S), N-cadherin (13116S), E-cadherin (3195S), Vimentin (5741S), Fibronectin (26836S), pan-keratin (4545T), Twist1 (69366S), SMAD3 (9523S), p-SMAD3 (9520T) and Slug (9585S) were purchased from Cell Signaling Technology (Danvers, MA, USA). Antibodies such as β-actin (sc-47778), α-Tubulin (sc-73242) and Notch1 (sc-376403) were purchased from Santa Cruz Biotechnology (Dallas, TX, USA). Antibodies EEA1 (ab70521) and LAMP1 (ab25631) were from Abcam, and Notch1 (AF1546), Ubiquitin (AF1705) and Histone3 (AF0009) from Byotime (Nantong, China).

### 4.2. siRNAs, Plasmids, Generation of Viruses and Stable Cell Lines

Different siRNA oligos were synthesized from Gene Pharma (Shanghai, China). Cells were transfected with siRNA oligos using Lipofectamine RNAiMAX (Invitrogen, Carlsbad, CA, USA). 3XFlag-N1ICD overexpression plasmids were purchased form Addgene. NUMB isoform (p65, p66, p71, or p72) expression plasmids and SMAD3-HA overexpression plasmids were generated based on the PCDNA3.0 vector. sh-p65/p66-#1/#2, sh-p71/p72-#1/#2 and shNC lentivirus were generated with PLKO.1-puro lentiviral vector in 293T cells. RKO and HeLa cells were infected with lentivirus and selected for puromycin resistance. The siRNA sequences used are listed in Supplementary Table S1, primer sequences for plasmid construction are listed in Supplementary Table S2, shRNA sequences used are listed in Supplementary Table S3 and primer sequences for detecting NUMB specific isoforms are listed in Supplementary Table S4.

### 4.3. Cell Migration Assay

The cell migration assay was performed using modified Boyden chambers in 24-well dishes with Transwell filter inserts provided with 8 μm pore membranes (Corning Inc., Corning, NY, USA). Cells were seeded into each upper chamber of the insert in serum-free medium, and 10% fetal bovine serum was added to the lower chamber. After 24 or 48 h, cells were fixed with 4% paraformaldehyde and stained using 0.1% crystal violet. Cells in the upper chamber were carefully removed, and the cells that migrated through the lower side of the filter were imaged (Olympus IX81) and quantified with ImageJ.

### 4.4. Ethics Statement 

All animal experiments were performed in accordance with a protocol approved by the Institutional Animal Care and Use Committee of Shanghai Institute of Nutrition and Health, the Chinese Academy of Sciences. The project identification code is SINH-2021-FY-1, and the approval date is 1 June 2021.

### 4.5. Xenograft Assay in Nude Mice

Four -week-old male nude mice were purchased from the Shanghai Experimental Animal Center (Shanghai, China) and maintained under pathogen-free conditions. Nude mice were injected subcutaneously on the right flank with tumor cells (3 × 10^6^ cells/mouse) suspended in 100 μL serum-free medium and mixed 2:1 (*v*/*v*) with Matrigel. After four weeks, the mice were sacrificed, and tissues including livers, lungs and spleens were harvested from the nude mice for further analysis.

### 4.6. Immunofluorescence Staining and HE Staining

Indicated cells were grown to medium density on glass coverslips. At 24 h after adherence, the cells were fixed with 4% paraformaldehyde for 15 min. After permeabilization in 0.05% Triton X-100 for 10 min and 5% BAS for 60 min, cells were then incubated with primary antibodies at 4 °C overnight, followed by PBS washes 3 times, and incubated with secondary antibodies. The nucleus of the cells was stained with DAPI. The glass slides were examined using a confocal microscope, and representative fields were photographed. Mice xenografts were formalin-fixed, paraffin-embedded, and sectioned (5 mm). Samples were stained with hematoxylin and eosin (H&E) or with the indicated antibodies.

### 4.7. Western Blotting and Co-Immunoprecipitation

Western blotting and co-immunoprecipitation were all carried out as previously described [25]. Briefly, 293T cells were co-transfected with indicated plasmids for 48 h. Then, cells were lysed in immunoprecipitation (IP) lysis buffer (20 mM Tris–HCl (pH 7.5), 100 mM NaCl, 1 mM EDTA, 10% glycerol, 0.1% NP-40 and 1% Triton X-100) and protease inhibitor mixture. Co-IP was performed with the indicated antibody for 4 h at 4 °C. Immunoprecipitations were conducted at 4 °C overnight using protein A/G beads (Santa Cruz Biotechnology). The samples were washed 4 times with IP lysis buffer, and beads were taken up in the SDS Laemmli buffer and heated for 5 min at 95 °C. Samples were loaded on SDS-PAGE gels, followed by Western blotting. 

### 4.8. In Vivo Transportation Assay

Cells were coated on glass plates and transfected with indicated siRNAs or plasmids for 48 h, then replaced with cold 10% FBS and incubated at 4 °C for 30 min to block the processing of cellular vesicular transport. Then, they were replaced with 37 °C pre-warmed culture medium and incubated at 37 °C for 15 min to re-activate the vesicular transportation. Cells were fixed with 4% paraformaldehyde for 15 min, followed by permeabilization in 0.05% Triton X-100 for 10 min and 5% BAS for 60 min, and were subjected to immunostaining with indicated antibodies. Images were produced using a confocal laser scanning microscope (LSM880).

### 4.9. Statistical Analyses

All experiments were repeated at least three times. All data presented as histograms refer to mean values ± standard deviation (SD) of the total number of independent experiments. Statistical analysis was performed by Student’s *t* test, and *p* values of * *p* < 0.05, ** *p* < 0.01 and *** *p* < 0.001 were considered to be statistically significant.

## Supplementary Materials

The following supporting information can be downloaded at: https://www.mdpi.com/article/10.3390/ijms23084363/s1.
