# Supplementary material for: Exclusion of NUMB Exon12 Controls Cancer Cell Migration through Regulation of Notch1-SMAD3 Crosstalk
Source: Int J Mol Sci. 2022 Apr 14;23(8):4363. doi: 10.3390/ijms23084363 (PMC9027642; doi:10.3390/ijms23084363)
Supplement: Supplementary file 1 [file ijms-23-04363-s001.zip › ijms-1670453-supplementary.pdf]

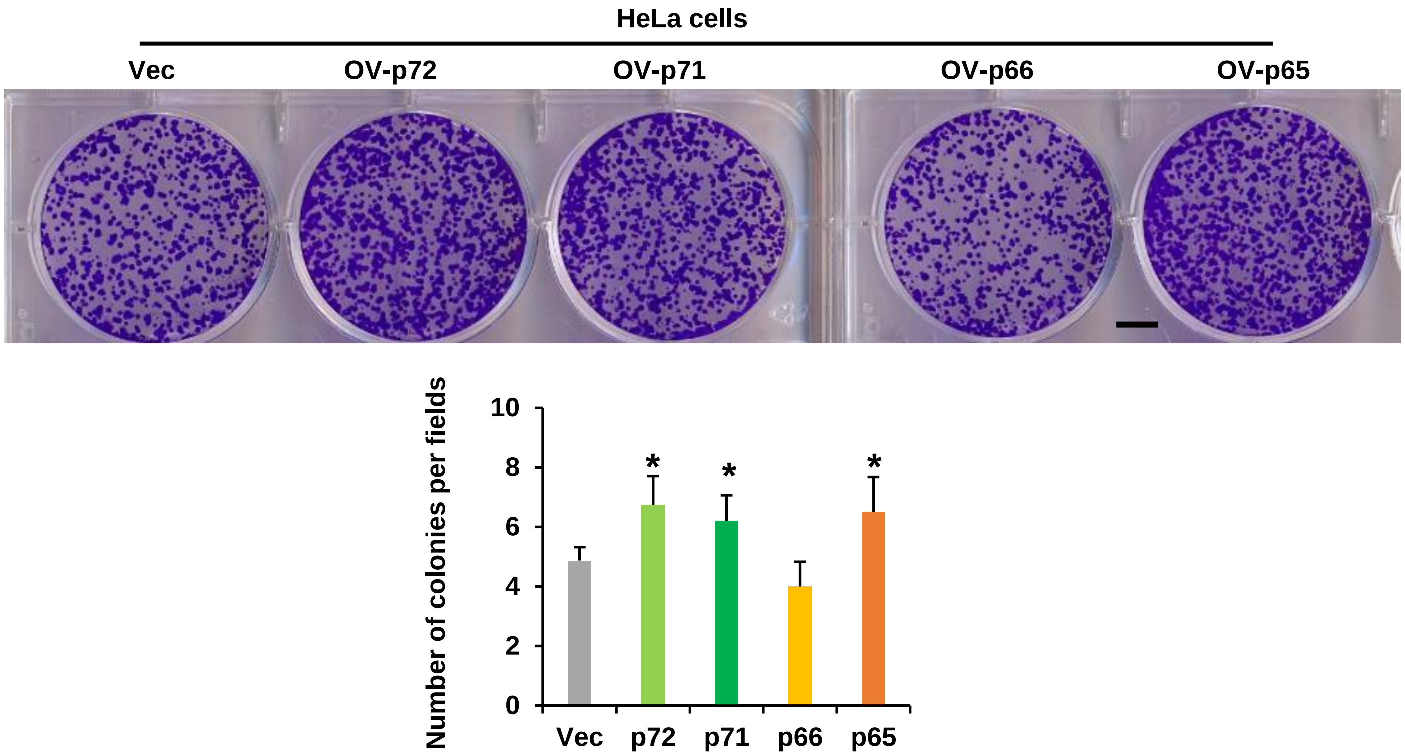

Supplementary Figure S1. NUMB isoforms including p72, p71 and p65 promoted cancer cell proliferation. Clonogenic survival assays were performed using cells described in Fig. 1B. The relative number of focal adhesions was quantified in the bar graph below. Scale bar is 500 um.

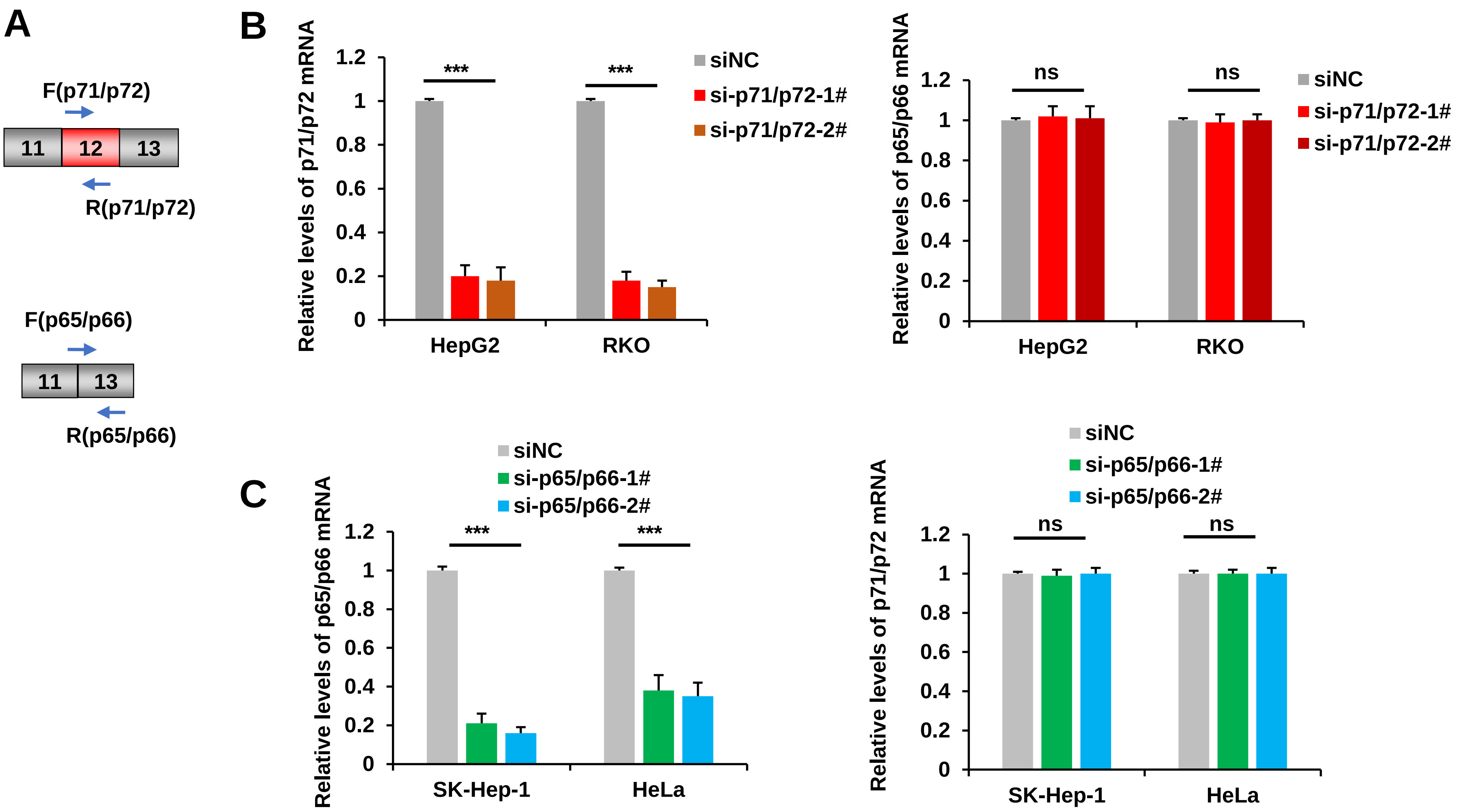

Supplementary Figure S2. Knockdown specificity of NUMB isoforms examined by RT-qPCR. (A) Illustration of primers used to specifically detect NUMB p71/p72 or NUMB p65/p66 variants. (B) HepG2 and RKO cells were transiently transfected with si-RNAs against p71/p72, followed by RT-qPCR analysis. Relative mRNA levels of p71/p72 or p65/p66 isoforms were shown, respectively. C. SK-Hep-1 and HeLa cells were transiently transfected with si-RNAs against p65/p66, followed by QT-qPCR analysis. Relative mRNA levels of p65/p66 or p71/p72 were shown, respectively.

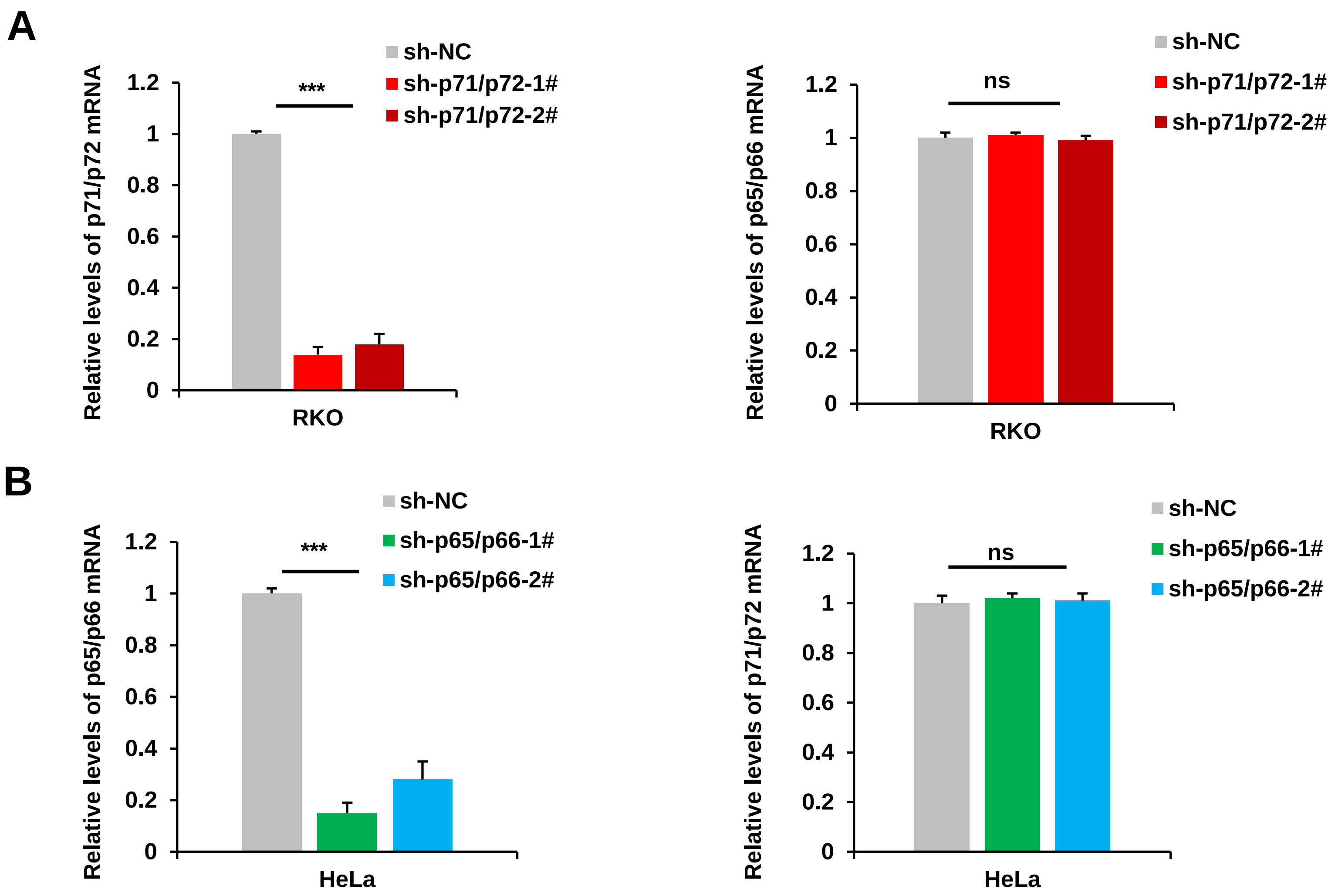

Supplementary Figure S3. Knockdown specificity of NUMB isoforms examined by RT-qPCR. (A) Relative mRNA levels of NUMB isoforms were compared between stable RKO cells expressing sh-p71/p72(1#, or 2#) and sh-NC expressing cells. (B) Relative mRNA levels of NUMB isoforms were compared between stable HeLa cells expressing sh-p65/p66(1# or 2#) and sh-NC expressing cells.

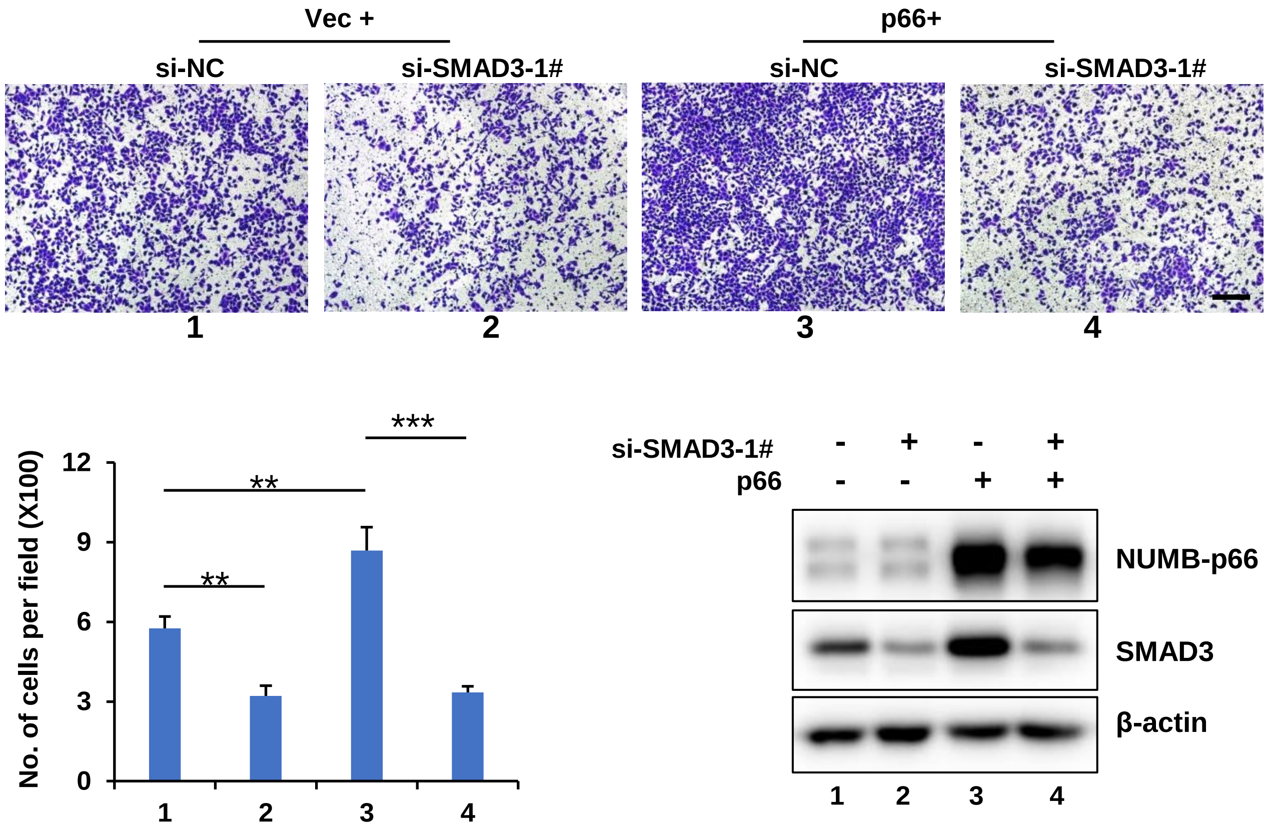

Supplementary Figure S4. SMAD3 knockdown rescue the migration advantage seen in the NUMB-p66 overexpression. HeLa cells were co-transfected with si-SMAD3 siRNA and NUMB p66 plasmid as indicated, followed by the cell migration assay. Representative images of migratory cells stained with crystal violet were shown on the top. Scale bar is 200 um. Quantification of migratory cells as mean ± SD was shown on the left bottom. Western blot analysis was shown on the right bottom.

Supplementary Table S1. siRNA sequences used for RNA interference.

siRNA	Sense(5'-3')	Antisense(5'-3')
si-NC	UUCUCCGAACGUGUCACGUTT	ACGUGACACGUUCGGAGAATT
si-NUMB-p71/p72-#1	CCCUGAUGCUGCUAACAAGTT	CUUGUUAGCAGCAUCAGGGTT
si-NUMB-p71/p72-#2	CACUGACUCAGCCUUCCAUTT	AUGGAAGGCUGAGUCAGUGTT
si-NUMB-p65/p66-#1	ACCUUCCAAGGGACCGAGUTT	ACUCGGUCCCUUGGAAGGUTT
si-NUMB-p65/p66-#2	CCUUCCAAGGGACCGAGUGTT	CACUCGGUCCCUUGGAAGGTT
si-SMAD3-1#	CUCCAAUGUCAACAGGAAUTT	AUUCCUGUUGACAUUGGAGTT
si-SMAD3-2#	CUCCUACUACGAGCUGAACTT	GUUCAGCUCGUAGUAGGAGTT
si-Notch1	CCAACUGCCAGACCAACAUTT	AUGUUGGUCUGGCAGUUGGTT

Supplementary Table S2. Primer sequences used for plasmid construction.

Gene	Sense(5'-3')	Antisense(5'-3')
SMAD3-HA	GCTCTAGAATGTCGTCCATCCTGCCTTTC	CGGAATTCCTAAGACACACTGGAACAGCG
SMAD3-Flag	CGGGATCCATGTCGTCCATCCTGCCTTTC	CGGAATTCCTAAGACACACTGGAACAGCG
NUMB-p65	GCTCTAGAATGAACAAATTACGGCAAAG	CCGGAATTCTTAAAGTTCAATTTCAAACG
NUMB-p66	GCTCTAGAATGAACAAATTACGGCAAAG	CCGGAATTCTTAAAGTTCAATTTCAAACG
NUMB-p71	GCTCTAGAATGAACAAATTACGGCAAAG	CCGGAATTCTTAAAGTTCAATTTCAAACG
NUMB-p72	GCTCTAGAATGAACAAATTACGGCAAAG	CCGGAATTCTTAAAGTTCAATTTCAAACG
HA-p65	CCGGAATTCCGATGAACAAATTACGGCAAAG	CCGCTCGAGTTAAAGTTCAATTTCAAACG
HA-p66	CCGGAATTCCGATGAACAAATTACGGCAAAG	CCGCTCGAGTTAAAGTTCAATTTCAAACG
HA-71	CCGGAATTCCGATGAACAAATTACGGCAAAG	CCGCTCGAGTTAAAGTTCAATTTCAAACG
HA-p72	CCGGAATTCCGATGAACAAATTACGGCAAAG	CCGCTCGAGTTAAAGTTCAATTTCAAACG

Supplementary Table S3. shRNA sequences used for RNA interference.

shRNA	Sense(5'-3')/ Antisense(5'-3')
sh-NC-F	ccggAATTCTCCGAACGTGTCACGTCTCGAGACGTGACACGTTCGGAGAATTTTTTTg
sh-NC-R	aattcAAAAAAATTCTCCGAACGTGTCACGTCTCGAGACGTGACACGTTCGGAGAATT
sh-p71/p72-#1-F	ccggAACCCTGATGCTGCTAACAAGCTCGAGCTTGTTAGCAGCATCAGGGTTTTTTTg
sh-p71/p72-#1-R	aattcAAAAAAACCCTGATGCTGCTAACAAGCTCGAGCTTGTTAGCAGCATCAGGGTT
sh-p71/p72-#2-F	ccggAACACTGACTCAGCCTTCCATCTCGAGATGGAAGGCTGAGTCAGTGTTTTTTTg
sh-p71/p72-#2-R	aattcAAAAAAACACTGACTCAGCCTTCCATCTCGAGATGGAAGGCTGAGTCAGTGTT
sh-p65/p66-#1-F	ccggAAACCTTCCAAGGGACCGAGTCTCGAGACTCGGTCCCTTGGAAGGTTTTTTTTg
sh-p65/p66-#1-R	aattcAAAAAAAACCTTCCAAGGGACCGAGTCTCGAGACTCGGTCCCTTGGAAGGTTT
sh-p65/p66-#2-F	ccggAACCTTCCAAGGGACCGAGTGCTCGAGCACTCGGTCCCTTGGAAGGTTTTTTTg
sh-p65/p66-#2-R	aattcAAAAAAACCTTCCAAGGGACCGAGTGCTCGAGCACTCGGTCCCTTGGAAGGTT

Supplementary Table S4. Primer sequences used for p71/p72 and p65/p66 qPCR analysis.

NUMB isoforms	Sense(5'-3')	Antisense(5'-3')
p71/p72	CTAATGGCACTGACTCAGCC	CCTTGTTAGCAGCATCAGGG
p65/p66	CCTTCCAAGCTAATGGCACTG	CTTGTTAGCAGCATCAGGGG
